# Impact of Bevacizumab treatment intervals on surgical interventions in pediatric neuro-oncology

**DOI:** 10.1093/noajnl/vdag073

**Published:** 2026-03-21

**Authors:** Hannah Schned, Maria Aliotti Lippolis, Cora Hedrich, Natalia Stepien, Abed Zodjad Ahmadi, Alicia Baumgartner, Oliver Eichmueller, Thomas Czech, Irene Slavc, Martin Lothar Metzelder, Karl Roessler, Lisa Mayr, Christian Dorfer, Amedeo Arthur Azizi, Andreas Peyrl, Johannes Gojo

**Affiliations:** Department of Pediatrics and Adolescent Medicine, Medical University of Vienna, Vienna, Austria; Comprehensive Cancer Center, Medical University of Vienna, Vienna, Austria; Comprehensive Center for Pediatrics, Medical University of Vienna, Vienna, Austria; Department of Pediatrics and Adolescent Medicine, Medical University of Vienna, Vienna, Austria; Comprehensive Cancer Center, Medical University of Vienna, Vienna, Austria; Comprehensive Center for Pediatrics, Medical University of Vienna, Vienna, Austria; Department of Pediatrics and Adolescent Medicine, Medical University of Vienna, Vienna, Austria; Comprehensive Cancer Center, Medical University of Vienna, Vienna, Austria; Comprehensive Center for Pediatrics, Medical University of Vienna, Vienna, Austria; Department of Pediatrics and Adolescent Medicine, Medical University of Vienna, Vienna, Austria; Comprehensive Cancer Center, Medical University of Vienna, Vienna, Austria; Comprehensive Center for Pediatrics, Medical University of Vienna, Vienna, Austria; Department of Pediatrics and Adolescent Medicine, Medical University of Vienna, Vienna, Austria; Comprehensive Cancer Center, Medical University of Vienna, Vienna, Austria; Comprehensive Center for Pediatrics, Medical University of Vienna, Vienna, Austria; Department of Pediatrics and Adolescent Medicine, Medical University of Vienna, Vienna, Austria; Comprehensive Cancer Center, Medical University of Vienna, Vienna, Austria; Department of Pediatrics and Adolescent Medicine, Medical University of Vienna, Vienna, Austria; Comprehensive Cancer Center, Medical University of Vienna, Vienna, Austria; Comprehensive Center for Pediatrics, Medical University of Vienna, Vienna, Austria; Comprehensive Cancer Center, Medical University of Vienna, Vienna, Austria; Comprehensive Center for Pediatrics, Medical University of Vienna, Vienna, Austria; Department of Neurosurgery, Medical University of Vienna, Vienna, Austria; Department of Pediatrics and Adolescent Medicine, Medical University of Vienna, Vienna, Austria; Comprehensive Cancer Center, Medical University of Vienna, Vienna, Austria; Comprehensive Center for Pediatrics, Medical University of Vienna, Vienna, Austria; Comprehensive Cancer Center, Medical University of Vienna, Vienna, Austria; Comprehensive Center for Pediatrics, Medical University of Vienna, Vienna, Austria; Department of Pediatric and Adolescent Surgery, Medical University of Vienna, Vienna, Austria; Comprehensive Cancer Center, Medical University of Vienna, Vienna, Austria; Comprehensive Center for Pediatrics, Medical University of Vienna, Vienna, Austria; Department of Neurosurgery, Medical University of Vienna, Vienna, Austria; Department of Pediatrics and Adolescent Medicine, Medical University of Vienna, Vienna, Austria; Comprehensive Cancer Center, Medical University of Vienna, Vienna, Austria; Comprehensive Center for Pediatrics, Medical University of Vienna, Vienna, Austria; Comprehensive Cancer Center, Medical University of Vienna, Vienna, Austria; Comprehensive Center for Pediatrics, Medical University of Vienna, Vienna, Austria; Department of Neurosurgery, Medical University of Vienna, Vienna, Austria; Department of Pediatrics and Adolescent Medicine, Medical University of Vienna, Vienna, Austria; Comprehensive Cancer Center, Medical University of Vienna, Vienna, Austria; Comprehensive Center for Pediatrics, Medical University of Vienna, Vienna, Austria; Department of Pediatrics and Adolescent Medicine, Medical University of Vienna, Vienna, Austria; Comprehensive Cancer Center, Medical University of Vienna, Vienna, Austria; Comprehensive Center for Pediatrics, Medical University of Vienna, Vienna, Austria; Department of Pediatrics and Adolescent Medicine, Medical University of Vienna, Vienna, Austria; Comprehensive Cancer Center, Medical University of Vienna, Vienna, Austria; Comprehensive Center for Pediatrics, Medical University of Vienna, Vienna, Austria

**Keywords:** bevacizumab, children, CNS tumor, surgery, wound healing

## Abstract

**Background:**

Bevacizumab (BVZ) is widely used in patients with central nervous system (CNS) tumors. Due to its potential to impair wound healing, a minimum interval of 28 days between BVZ administration and surgery - both before and after the procedure - is generally recommended. However, strict adherence to this interval is often challenging in clinical practice, particularly when urgent oncologic treatment or time-sensitive surgical interventions are required.

**Methods:**

Pediatric patients with a CNS tumor receiving intravenous BVZ from January 2005 to December 2022 treated at the Medical University of Vienna were retrospectively analyzed for surgical complications.

**Results:**

One hundred and nineteen patients had 344 surgeries with BVZ therapy before and or after surgery. 11 wound complications (3.20%) of any grade (*n*=1 CTCAE grade I; *n*=10 CTCAE grade III) occurred in 11 children (9.24%). Group comparison of BVZ exposure intervals (BVZ ≤28/>28 days and ≤14/>14 days) revealed no statistically significant differences in wound complication rates, with odds ratios of 3.91 (≤28/>28 d; *P* = .55) and 1.15 (≤14/>14 d; *P*=1.00) before surgery as well as 1.93 (≤28/>28 d; *P* = .46) and 3.16 (≤14/>14 d; *P* = .11) after surgery.

**Conclusion:**

BVZ can be administered within ≤ 28 days before or after surgery when postponing the procedure is not feasible or when immediate (re)initiation of therapy is required, provided that non-absorbable sutures are used, and close wound-monitoring is ensured.

Key Points

- In the majority of cases BVZ can be administered ≤ 28 prior to and after surgical procedures.
- Intervals ≤ 28 days before and ≤ 14 days after surgery are correlated with a slightly higher risk to develop wound complications.
- Non-absorbable sutures should be used to allow precise control over timing of suture removal.

Importance of the Study

In this study, we retrospectively characterized the impact of shorter treatment intervals of Bevacizumab (BVZ), a recombinant humanized monoclonal antibody inhibiting the vascular endothelial growth factor (VEGF), on wound healing after surgical interventions. According to the manufacturer’s guidelines, BVZ treatment should be paused for at least 28 days pre- and postoperatively to prevent wound-healing complications. Although widely administered, only sparse data exist about complications in pediatric tumor patients requiring surgery. In addition, the suggested four-week-period can lead to a delay of important therapeutical interventions, so possible complications of BVZ therapy must be weighed against a delay in necessary oncologic treatment. Based on the rate and type of adverse events reported in this study, decreasing the proposed time interval to ≤ 28 days may be considered case-by-case with intense wound-monitoring, usage of non-absorbable sutures and with low-threshold surgical consultation.

Brain tumors are the most common tumor type in children (0-14 years), with an average annual age-adjusted incidence rate (AAAIR) of 5.61 per 100,000 population and the second most common tumor site among adolescents and young adults (15-38 years) with an AAAIR of 12.07 per 100,000 population in the United States.^1^ Treatment of pediatric brain tumors, depending on type of tumor, is complex and involves a multidisciplinary approach including surgery, chemotherapy, radiotherapy and, increasingly, targeted or antiangiogenic therapies.^2-8^

The use of novel antiangiogenic agents has entered clinical practice since the first approval of the vascular endothelial growth factor (VEGF) inhibitor Bevacizumab (BVZ), a recombinant humanized monoclonal antibody inhibiting VEGF, by The United Stated Food and Drug Administration (FDA) in 2004 and The European Medicines Agency (EMA) in 2005 for various cancer types (eg colorectal cancer, breast cancer, lung cancer, kidney cancer, ovarian cancer). BVZ use has increased significantly in recent years and studies have proven its efficacy also in patients with pediatric central nervous system (CNS) tumors.^7^^,^^9-17^

Sustained angiogenesis with the development of new blood vessels is a hallmark of cancer and ensures a sufficient nutrient and oxygen supply for tumor cells. Tumor growth is characterized by an overexpression of VEGF, provided by different cells in the body, such as endothelial cells, platelets, macrophages, neutrophils, monocytes, consequently leading to formation, enlargement and even morphological modifications of blood vessels. The signaling pathway is therapeutically interfered with by the high-affinity binding of BVZ to VEGF further inhibiting the attachment to its VEGF-receptor expressed on the surface of endothelial cells in blood vessels.^18-20^ In addition, BVZ is often used for its anti-edema effects and to treat radiation-induced adverse events.^21^

Observed adverse events of BVZ include leukopenia, thrombocytopenia, anemia, proteinuria, hypertension, tachycardia, headache, dyspnea, peripheral neuropathy, thrombosis, gastrointestinal perforation, epistaxis, diarrhea, and caution needs to be warranted when administering BVZ in a perioperative setting as it is associated with an increased risk of impaired wound healing, infection and bleeding.^19^^,^^22^^,^^23^ Wound healing itself is a complex cellular, humoral and molecular process ranging from an inflammatory phase to a proliferative and finally a remodeling phase. During proliferation, which is driven by VEGF among other factors, released enzymes enable tissue repair.^24-26^

In the context of oncological surgical procedures, preventing treatment-delaying complications such as bleeding, wound infections or wound dehiscence is critical. With a reported half-life of approximately 18 days in female and 20 days in male patients, it is recommended to withhold BVZ therapy for at least 28 days before and after surgical interventions. This interval may need to be extended if wound healing remains incomplete postoperatively.^27^ Similar pharmacokinetic profiles have been documented in pediatric populations, with younger children demonstrating the longest observed half-life.^28-30^

Especially in pediatric neuro-oncology, omitting BVZ for the proposed interval often conflicts with the urgent need of systemic therapy or surgical interventions, as high-grade brain tumors may substantially progress within this period. Acute interventions such as placement of a ventriculoperitoneal shunt (VP-shunt) to treat hydrocephalus or placement of an Ommaya reservoir to initiate intraventricular treatment of leptomeningeal metastases may therefore be urgently needed. As a consequence, the interval to postsurgical re-administration must be as short as possible to minimize treatment delay, but as prolonged as needed, without causing BVZ-associated complications. Yet, little evidence exists on the risk of shorter intervals between surgical interventions and BVZ administration in pediatric brain tumor patients. In the present study, we investigated BVZ management and associated surgical complications in a single center cohort of pediatric CNS tumor patients.

## Methods

All patients aged 0 to 18 years at the time of diagnosis with a central nervous system tumor who received intravenous (IV) BVZ as part of their oncologic therapy from January 2005 to December 2022 at the Department of Pediatrics and Adolescent Medicine (Division of Neonatology, Intensive Care Medicine and Neuropediatrics, Medical University of Vienna) were included in the study. The study was approved by the local Ethics Committee of the Medical University of Vienna (ECS 1947/2020).

Bevacizumab administration at our institution is guided by both protocol-defined indications and individualized clinical judgment. Beyond its use within established treatment regimens, BVZ is selectively applied in patients with cystic tumor components, substantial peritumoral edema, or in conjunction with radiotherapy to reduce steroid exposure and improve symptomatic control.

Intravenous BVZ was routinely administered bi- or three-weekly in a dose range of 5 to 15 mg/kg (the far most common dosing being bi-weekly 10 mg/kg), and adjusted if adverse events occurred. All pediatric neuro-oncological patients were screened for any type of surgery during treatment, including external surgeries.

Surgeries were categorized into neurosurgical or general/non-neurosurgical interventions and according to their degree of invasiveness. Four groups were defined as followed: (1) minor non-neurosurgery (such as implantation of a central venous line; eg Broviac^®^ catheter, Hickmann^®^ catheter or Port-a-Cath [PAC]); (2) major non-neurosurgery (eg tumor resection); (3) minor neurosurgical intervention (eg implantation of a VP-shunt or an Ommaya-reservoir); and (4) major neurosurgical intervention (eg CNS tumor resection). Observed complications (postoperative bleeding, wound dehiscence, reddening of the wound, liquor fistula, pus formation, wound healing problems of any other kind) were further investigated with special regard to the time interval to previous surgeries and to the preceding and following BVZ therapy. Information was received from doctor’s letters, nursing reports, outpatient reports from the neuro-oncology, neurosurgery, pediatric surgery department as well as from the surgical reports. Adverse events were classified according to the Common Terminology Criteria for Adverse Events criteria (CTCAE, Version 5, 2017).^31^

The time point of IV administration of BVZ prior and after surgery with the exact dosage and number of administrations were documented. Several time-intervals between a surgical intervention and therapy with BVZ were defined: ≤ 28 days (cut-off point from manufacturer and literature) compared to > 28 days as well as ≤ 14 vs. > 14 days (time span occasionally employed at our clinic to avoid postponing urgently needed surgery or continuation of systemic therapy).

To already minimize tissue damage during surgery, monopolar cauterization and excessive bipolar coagulation are not employed during incision as part of our local surgical policy. Particular care is further taken to prevent tension on the wound margins during surgery, especially in case of long surgery duration. Non-resorbable materials are generally used for skin closure to allow precise control over timing of suture removal, particularly when Bevacizumab administration is planned.

After surgery a close monitoring of the wound is meticulously performed by experienced pediatric neuro-oncologists, pediatric neurosurgeons, pediatric and adolescent surgeons as well as nurses.

## Results

From January 2005 to December 2022, 171 patients received intravenous BVZ as an oncologic therapy, of whom 119 patients had at least one surgical intervention following or preceding BVZ treatment.

Median patient age at time point of surgery was 8.55 years (range two months to 25 years) with different underlying diagnoses, medulloblastoma making up the largest fraction (29%, *n*=35) followed by low-grade glioma (20%, *n*=24; *n*=22 pilocytic astrocytoma, n=1 pleomorphic xanthoastrocytoma and *n*=1 diffuse low-grade glioma), ependymoma (13%, *n*=15), and high-grade glioma (12%, *n*=14; including *n*=11 diffuse midline glioma, H3K27-altered, *n*=2 glioblastoma, IDH-wildtype, n=1 diffuse pediatric-type high-grade glioma, H3-wildtype and IDH-wildtype) and other entities (see Table 1, Table S1, and Figure S1).

**Table 1.** Baseline characteristics

Parameter	Unit	Amount
Total number of patients receiving BVZ	n	171
Total number of patients receiving BVZ and a surgical intervention	n (%)	119/171 (69.59)
Median (range) age in years (y) at surgery	y	8.55 (2 months, 25 y)
Gender (f)	n (%)	53/119 (44.54)
Surgeries in total	n (%)	344
Minor non-neurosurgery (eg implantation of a Broviac catheter)	n (%)	136/344 (39.53)
Major non-neurosurgery (eg pancreatic resection)	n (%)	9/344 (2.62)
Minor neurosurgical interventions (eg Ommaya implantation)	n (%)	120/344 (34.88)
Major neurosurgical interventions (eg tumor resection)	n (%)	79/344 (22.97)

BVZ = Bevacizumab; f = female; n = number; y = years; % = percentage.

Overview of baseline characteristics of the study cohort, including patient age, sex, and types of surgical interventions performed in the context of Bevacizumab therapy.

A total of 344 surgical interventions was performed. Of these, the majority were minor non-neurosurgical procedures (*n*=136/344, 39.53%). Major non-neurosurgical procedures accounted for 2.62% of interventions (*n*=9/344), while 34.88% were minor neurosurgical (*n*=120/344) and 22.97% major neurosurgical procedures (*n*=79/344) (see Table 2; Table S2). Radiotherapy was performed in 21.51% of patients (*n*=74/344), either at the time of surgery or within one month perioperatively. Corticosteroid treatment (dexamethasone) was given concurrently with BVZ in four out of 119 patients (3.36%).

**Table 2.** Complications

Parameter	Amount	CTCAE grading
Wound complications in total	*n*=11/344 (3.20 %)	
Patients affected by a wound complication	*n*=11/119 (9.24 %)	
Revision surgery due to complication	*n*=10/11 (90.91 %)	
Local therapy (eg local wound disinfection, secondary suture)	*n*=1/11 (9.09 %)	
Minor non-neurosurgical interventions preceding complications	*n*=3/11 (27.27 %)	III: *n*=3/11 (27.27 %)
Major non-neurosurgical interventions preceding complications	*n*=1/11 (9.09 %)	III: *n*=1/11 (9.09 %)
Minor neurosurgical interventions preceding complications	*n*=3/11 (27.27 %)	I: *n*=1/11 (9.09 %) III: *n*=2/11 (18.18 %)
Major neurosurgical interventions preceding complications	*n*=4/11 (36.36 %)	III: *n*=4/11 (36.36 %)

	Mean		n/median/range/SD/CI 95 % []		*P*
	≤ 28 d	> 28 d	≤ 28 d	> 28 d	
Mean interval of BVZ before surgery ≤ 28/>28 days (d)	14.27 d	122.51 d	84/14/0:28/6.94/[12.77; 15.78]	45/74/29:351/99.65/[92.57; 152.45]	
Mean interval of BVZ after surgery ≤ 28/>28 d	12.15 d	103.73 d	200/11/0:28/6.71/[11.21; 13.09]	59/59/29:360/91.26/[79.95; 127.51]	
Mean dosage of BVZ before surgery ≤ 28/>28 d	9.88 mg	10.00 mg	84/10/5:10/0.77/[9.71; 10.05]	45/10/5:15/1.07/[9.68; 10.32]	.47
Mean dosage of BVZ after surgery ≤ 28/>28 d	9.90 mg	10.08 mg	200/10/5:10/0.70/[9.80; 10.00]	59/10/10:15/0.65/[9.92; 10.25]	.07
Mean number of BVZ administrations before surgery ≤ 28/>28 d	16.27	21.02	84/12/1:55/14.11/[13.21; 19.34]	45/14/1:69/17.44/[15.78; 26.26]	.13
Mean number of BVZ administrations after surgery ≤ 28/>28 d	6.27	6.97	200/1/1:56/11.55/[4.66; 788]	59/1/1:70/13.77/[3.38; 10.55]	.94

BVZ = Bevacizumab; CI 95% [] = 95% confidence interval, CTCAE = Common Terminology Criteria for Adverse Events; d = days; mg = milligram; n = number; *P*=*P*-value; SD = standard deviation; % = percentage.

Overview of surgical wound complications with CTCAE grading, alongside detailed timing, dosage and intervals of Bevacizumab therapy before and after surgical interventions.

BVZ was administered with a mean time difference of 14.27 days (group ≤ 28 days, *n*=84/344) and 122.51 days (group > 28 days, *n*=45/344) before surgery. After surgical procedures, therapy was administered with a mean time interval of 12.15 days (group ≤ 28 days, *n*=200/344) and 103.72 days (group > 28 days, *n*=59/344), respectively. Both before and after surgery, there were no statistically significant differences in dosage (*P* = .47; *P* = .07) as well as number of BVZ administrations (*P* = .13; *P* = .94) comparing groups ≤ 28 days and > 28 days. For non-resorbable sutures, removal was performed after a mean of 41.64 days, as a prolonged suture retention strategy was routinely applied in the context of planned BVZ treatment (median: 37 days; standard deviation [SD]: 25.18; range: 10-124 days; CI 95% [36.62; 46.66]).

Overall, 11 wound complications occurred among 344 reported surgical interventions (*n*=11/344, 3.20%), affecting 11 patients (*n*=11/119, 9.24%). Three minor non-neurosurgical (*n*=3/11, 27.27%), one major non-neurosurgical (*n*=1/11, 9.09%), three minor neurosurgical (*n*=3/11, 27.27%) and four major neurosurgical interventions (*n*=4/11, 36.36%) preceded the wound complications. Complications occurred with a median of 45 days after surgical procedures (mean: 49.9 days; SD: 9.79; range 6-106 days; CI 95% [28.1; 72.7]). Of the documented wound reactions, one required only local therapy (*n*=1/11, 9.09%) and 10 an additional surgical procedure in the operation room (*n*=10/11, 90.91%). Among the four patients who received concurrent corticosteroid treatment, one experienced a wound complication.

### Presurgical Administration

Within the group receiving BVZ ≤ 28 days before surgery (*n*=84/344), three complications (*n*=3/84, 3.57%) occurred in three patients after a median of 31 days (mean: 46.67 days; SD: 28.88; range: 29-80 days; CI 95% [−25.09; 118.42]), including one localized wound infection with slight redness after VP-shunt implantation needing regular local disinfection (CTCAE I); a CTCAE grade III episode of pus formation requiring drainage was observed after pancreatic resection, developing in the setting of a non-healing wound; and one local wound dehiscence after brain tumor resection requiring revision surgery in the operation room occurred (CTCAE III). Notably, in two cases resorbable sutures were used during first operation (*n*=2/3, 66.67%), in the case with non-resorbable sutures only minor local wound management was required. Two of the affected patients also received BVZ after initial surgery (*n*=2 within 28 days, *n*=1>28 days afterwards). In patients with BVZ > 28 days before surgery, no complication occurred.

### Postsurgical Administration

Within the group receiving BVZ ≤ 28 days after surgery (*n*=200/344) nine complications (*n*=9/200, 4.5%) occurred in nine patients after a median of 45 days (mean: 48.67 days; SD: 34.03; range: 6-106 days; CI 95% [22.51; 74.83]), all of them being classified as CTCAE grade III. One patient also received BVZ ≤ 28 days before surgery. At initial surgery, resorbable sutures were used in five cases (*n*=5/9, 55.56%), non-resorbable sutures in four cases (*n*=4/9, 44.44%). In the group receiving BVZ > 28 days after surgery (*n*=59/344), one complication (*n*=1/59, 1.69%) occurred after 31 days (CTCAE III). The patient had already received BVZ ≤ 28 days before surgery and non-resorbable sutures were used at initial surgery.

### Comparison of Complication Rates

Wound complications occurred in three out of 84 cases in patients receiving BVZ ≤ 28 days before surgery (*n*=3/84, 3.57%), no patient was affected by a wound complication receiving BVZ > 28 days prior to surgery (Fisher’s exact test, *P* = .55). After surgery, nine complications in the group ≤ 28 days (*n*=9/200, 4.50%) and one complication > 28 days (*n*=1/59, 1.69%) occurred (Fisher’s exact test, *P* = .46) (see Table 3).

**Table 3.** Risk stratification of wound complications before and after surgery

Risk Stratification before surgery (≤/> 28 days; ≤/> 14 days)					Statistical results^a^
	Receiving BVZ (in days)	No complications	Complications	Total	
Group 1	≤ 28 (≤ 14)	*81 (43)*	*3 (1)*	*84 (44)*	OR 3.91 CI 95 % [0.20; 77.35] (≤ 14/> 14: OR 1.15 [0.15; 9.01]) RR 3.79 [0.20; 71.76] (≤ 14/> 14: RR 1.15 [0.16; 8.40]) *P* = .55 (≤ 14/> 14: *P*=1.00)
Group 2	> 28 (> 14)	45 (83)	0 (2)	45 (85)	
	Total	126	3	129	

Risk Stratification after surgery (≤/> 28 days; ≤/> 14 days)					Statistical results^a^
	Receiving BVZ (in days)	No complications	Complications	Total	
Group 1	≤ 28 (≤ 14)	191 (129)	9 (8)	200 (137)	OR 1.9 [0.34; 11.10] (≤ 14/> 14: OR 3.16 CI 95 % [0.76; 13.24]) RR 1.89 [0.3; 10.33] (≤ 14/> 14: RR 3.03 CI 95 % [0.76; 12.16]) *P = .*46 (≤ 14/> 14: *P* = .11)
Group 2	> 28 (> 14)	58 (120)	1 (2)	59 (122)	
	Total	249	10	259	

BVZ = Bevacizumab; CI 95% [] = 95% confidence interval; OR = Odds Ratio.

a ORs were calculated using the Haldane-Anscombe continuity correction in the presence of zero cells; *P*-values were obtained using two-sided Fisher’s exact test.

Risk stratification comparing surgical wound complication rates between Bevacizumab therapy ≤28/> 28 days and ≤14/>14 days before and after surgery, including relative risk (RR) and odds ratio (OR) with corresponding *P*-values.

Due to therapeutic pressure in case of hydrocephalus or massive progressive disease/urgent need to continue therapy after a surgical procedure, some patients received BVZ perioperatively even within 14 days or less. Complication rate was therefore screened with a cut-off of 14 days. There were no statistically significant group differences. One out of 44 BVZ therapies administered ≤ 14 days before surgery (*n*=1/44, 2.27%; complication after VP-shunt implantation with non-resorbable sutures) and two out of 85 BVZ ­therapies administered > 14 days (*n*=2/85, 2.35%) were ­associated with wound complications (after major non-neurosurgery and major neurosurgery, both with resorbable sutures) (Fisher’s exact test, *P*-value=1.00). BVZ was given ≤ 14 days in the majority of cases prior a minor non-neurosurgery (*n*=19/44, 43.18%), in 11.36% before a major non-neurosurgical intervention (*n*=5/44) as well as before 34.09% minor (*n*=15/44) and 11.36% major neurosurgical interventions (*n*=5/44), respectively.

After surgery, eight complications occurred in the group receiving BVZ ≤ 14 days (*n*=8/137, 5.84%), compared to two complications in the group receiving BVZ > 14 days (*n*=2/122, 1.64%) (Fisher’s exact test, *P* = .11). BVZ was initiated within 14 days in 43.80% of cases following minor non-neurosurgical procedures (n=60/137) and in 2.92% after major non-neurosurgical interventions (*n*=4/137). Among neurosurgical interventions, BVZ was started ≤ 14 days in 37.23% after minor (*n*=51/137) and in 16.06% after major procedures (*n*=22/137). Of the eight postoperative complications, 37.5% occurred after PAC implantation (*n*=3/8) and 37.50% after tumor resection (*n*=3/8), while 25% were observed following shunt implantation (*n*=2/8). Resorbable sutures were used in 62.50% of these cases (*n*=5/8).

**Figure 1.**
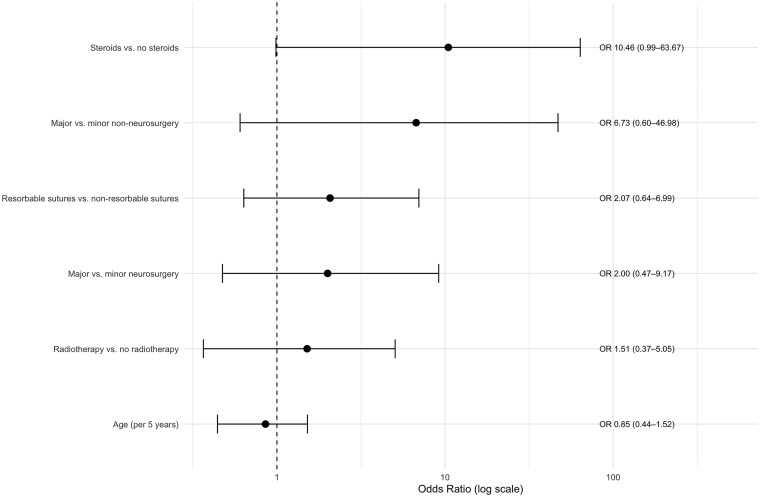
Logistic regression of predictors for surgical wound complications. Forest plot of univariable Firth logistic regression displaying the odds ratios (ORs) with 95% confidence intervals for potential predictors of postoperative wound complications, including age at surgery, surgery type (minor/major neurosurgical and non-neurosurgical procedures), perioperative radiotherapy exposure (≤ 1 month), steroid usage and suture material. *, Univariate Firth Logistic Regression including: steroid usage (steroid usage vs. no steroid usage; *P* = .05); surgery (major neurosurgery vs. minor neurosurgery (*P* = .34), major non-neurosurgery vs. minor non-neurosurgery (*P* = .11)); suture material (resorbable sutures vs. non-resorbable sutures; *P* = .22); radiotherapy (RTX) (RTX within 1 month perioperatively vs. no radiotherapy; *P* = .54); age at surgery per five-year increase (*P* = .61).

**Figure 2.**
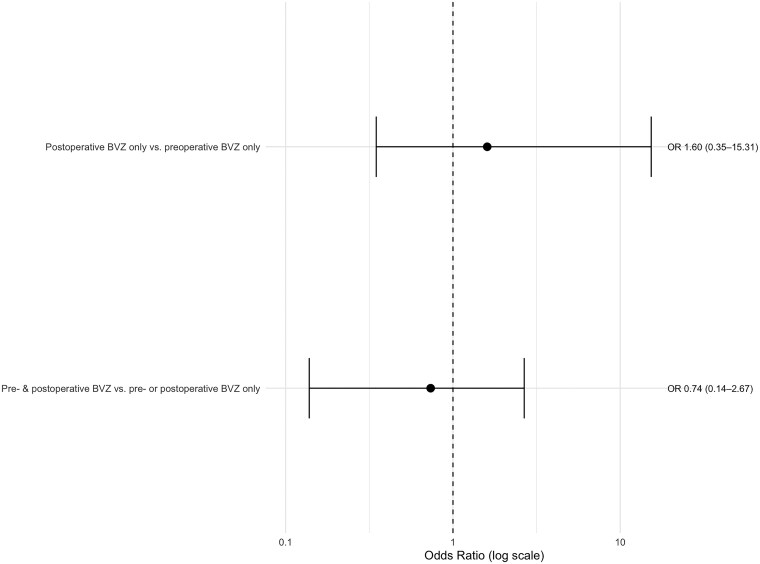

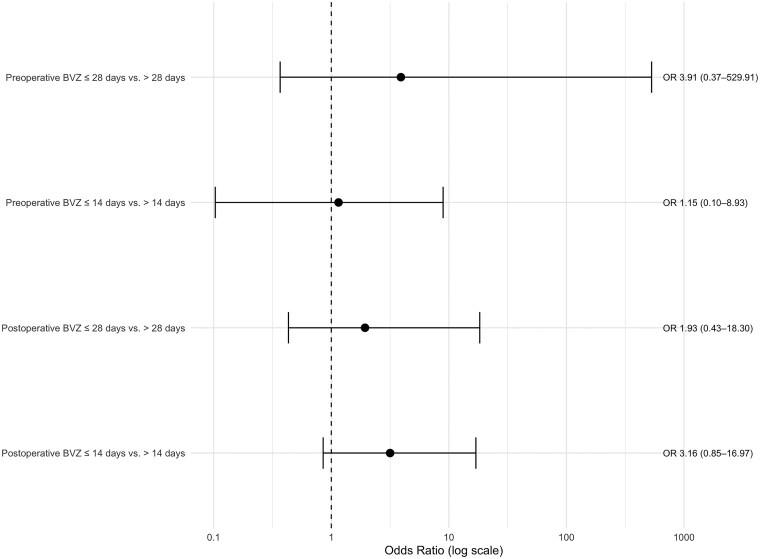
Association between timing of Bevacizumab administration and surgical complications. Two plots visualizing the odds ratios (ORs) and 95% confidence intervals (CI 95%) comparing (1) BVZ administration *before and after surgery* as well as (2) different BVZ timing strategies (≤ 14/> 14 days and ≤ 28/> 28 days). * Firth’s penalized Logistic Regression including timepoint of Bevacizumab (BVZ) administration. Left: BVZ Exposure. Postoperative BVZ only compared to preoperative BVZ only (*P* = .58); Pre- and postoperative BVZ compared to pre- or postoperative BVZ only (*P* = .66). Right: BVZ Timing. Preoperative BVZ administration ≤ 28/≤ 14 days compared to > 28/> 14 days (Preoperative BVZ ≤ 28 vs. > 28 days (*P* = .30); Preoperative BVZ ≤ 14 vs. > 14 days (*P* = .89); Postoperative BVZ ≤ 28 vs. > 28 days (*P* = .43); Postoperative BVZ ≤ 14 vs. > 14 days (*P* = .09)).

A univariate Firth logistic regression model was performed to assess the association between clinical and surgical factors and the occurrence of wound complications. Increasing age was not associated with a higher risk of wound complications, with an OR of 0.85 per additional five years (CI 95% [0.44; 1.52]; *P* = .61). Perioperative radiotherapy (within one month before or after surgery) showed an OR of 1.51 (CI 95% [0.37; 5.05]; *P* = .54). Compared with minor procedures, major neurosurgery was associated with an OR of 2.00 (CI 95% [0.47; 9.17]; *P* = .34), while major non-neurosurgery showed a higher OR of 6.73 (CI 95% [0.60; 46.98]; *P* = .11). The use of resorbable sutures was also associated with increased odds of wound complications (OR 2.07; CI 95% [0.64; 6.99]; *P* = .22). The highest odds were observed for steroid usage, with an OR of 10.46 (CI 95% [0.99; 63.67]; *P* = .05). Using Firth’s penalized logistic regression, postoperative BVZ exposure was associated with higher odds compared with preoperative administration alone (OR 1.60; CI 95% [0.35; 15.31]; *P* = .58), however, no increased risk was observed when BVZ was administered both pre- and postoperatively (OR 0.74; CI 95% [0.14; 2.67]; *P* = .66). Preoperative BVZ administration within 28 days was associated with higher odds of wound complications compared with administration more than 28 days prior to surgery (OR 3.91; CI 95% [0.37; 529.91], *P* = .30), whereas administration within 14 days did not show higher odds (OR 1.15; CI 95% [0.10; 8.93], *P* = .89). Postoperatively, administration within 28 days was associated with an odds ratio of 1.93 (CI 95% [0.43; 18.30]; *P* = .43), while administration ≤ 14 days showed higher odds (OR 3.16; CI 95% [0.85; 16.97)]; *P* = .09) (see Table 3, Figures 1 and 2).

## Discussion

Endogenous VEGF expression is significantly upregulated as a physiologic response to tissue damage induced by surgery, with peak wound tissue levels observed at two weeks post-surgery. These levels decrease by 28 days, but can remain detectable up until 24 weeks.^32^ As a potent inhibitor of VEGF, BVZ is known to impair wound healing in the perioperative setting.^19^^,^^23^ Nevertheless, BVZ remains a crucial component of various anti-cancer regimens, including those used in pediatric neuro-oncology.

In adult BVZ studies, particularly those involving colorectal and breast cancer metastases, wound healing complications have been reported in 1.7%-15% of patients.^33-35^ Brain tumor patients showed wound healing complications in 6.9%-35%.^36-38^

In pediatric patients, wound complications without BVZ occurred 6.7% after general surgeries and in 5.6% after neurosurgical procedures overall.^39^^,^^40^ Surgical site infections were observed in approximately 8% of cases following both shunt surgery and spinal tumor resection, respectively, as well as in 2% after brain tumor surgery.^41-43^ A phase II trial evaluating the addition of BVZ to standard osteosarcoma therapy reported impaired wound healing after primary tumor resection in approximately half of the patients (aged 6.8 to 20.3 years), leading to a discontinuation of 21% of the planned BVZ treatments.^44^ Similarly, studies on pediatric high-grade gliomas found that adding BVZ to radiotherapy and temozolomide was associated with a higher incidence of serious adverse events compared to radiotherapy and temozolomide alone (58% [*n*=35/60] vs. 48% [*n*=27/56] with 13 adverse events of special interest reported: proteinuria: 13% [*n*=8/60] vs. 0%, and arterial thromboembolic events: 8% [*n*=5/60] vs. 4% [*n*=2/56]).^45^ Other studies examining wound complication rates were only based on small patient cohorts, reporting rates ranging from 5 to 11.1%.^17^^,^^46-48^ Nevertheless, in otherwise healthy individuals, a perioperative cessation of BVZ for 28 days is generally considered safe to avoid delays in surgery or necessary therapy.^49^ Smaller surgeries should be avoided for at least 14 days.^50^^,^^51^

In this study, no statistically significant differences in wound complication rates were observed between patients receiving intravenous BVZ within 28 days and those with longer treatment interruptions exceeding 28 days, either before or after surgery. Comparable results were obtained for even shorter exposure intervals of ≤ 14 days. Although point estimates suggested higher odds of wound complications with shorter BVZ-to-surgery intervals—both preoperatively (OR 3.91 for ≤ 28 vs. > 28 days) and postoperatively (OR 3.16 for ≤ 14 vs. > 14 days)—these associations did not reach statistical significance.

Documented wound healing complications in our cohort were primarily minor. Surgical wound revisions were manageable without requiring further therapy or another intervention. Most importantly, wound management did not lead to substantial delays in the initiation or continuation of oncologic therapy, including radiotherapy.

In the majority of cases with wound complications absorbable suture material was used during initial surgery (*n*=6/11, 54.55%). Notably, all subsequent surgical revisions in these patients were performed using non-absorbable sutures, a strategy that appeared to mitigate the risk of recurrent wound dehiscence. Non-absorbable sutures allow for precise postoperative control and enable suture removal to be tailored to complete and definitive wound closure. Consistent with this, the use of absorbable sutures was associated with an increased risk of wound complications in our cohort (OR 2.07). Taken together, our findings suggest that employing non-absorbable sutures as a standard practice to allow precise control over timing of suture removal and even delaying suture removal until at least three weeks postoperatively may further reduce the risk of complications in patients requiring perioperative BVZ therapy, or particularly when further antiangiogenic therapy is required. This approach, along with intensified wound monitoring, likely contributed to the favorable outcomes observed.

Our study highlights the importance of balancing the risks of perioperative complications with the need to maintain timely oncological treatment. Delaying treatment to accommodate longer cessation periods for BVZ may have a more pronounced negative impact on oncological outcomes than the minor wound complications observed in our cohort. This underscores the necessity of individualized treatment planning, where the timing of BVZ-administrations and surgical intervention is optimized based on patient-specific factors and the urgency of oncological therapy.

It is worth noting, that minor surgical procedures, such as secondary suturing after wound dehiscence may not have been fully documented. This limitation may have led to an underestimation of low-grade wound complications. Nonetheless, the rates of wound healing due to BVZ treatment reported in the present analysis align with clinical experience and existing literature.^46^^,^^52^^,^^53^

The present analyses are further subject to limitations related to the low number of wound complications observed in the study cohort. The small number of outcome events resulted in wide 95% confidence intervals for several effect estimates, reflecting limited statistical precision and reduced power to detect modest associations. To mitigate potential bias arising from sparse data and zero-event cells, Firth’s penalized logistic regression was applied, which provides more stable and finite estimates under such conditions. Nevertheless, the width of the confidence intervals indicates that the absence of statistically significant associations for some comparisons does not preclude clinically relevant effects. Larger studies with higher event rates will be required to obtain more precise effect estimates and to further clarify the relationship between BVZ timing, surgical factors, and wound complications.

All included patients were treated at a single, high-volume pediatric neuro-oncology center with standardized perioperative protocols, extensive experience with complex (brain tumor) surgery, and strict wound-care pathways. These institutional factors likely contributed to optimized surgical conditions and may have reduced the overall incidence of wound-healing complications in our cohort. As a consequence, the observed complication rates may underestimate the true risk in settings with lower surgical volume, less specialized teams, or more heterogeneous perioperative management. Therefore, the external validity of our findings is limited, and caution is warranted when extrapolating these results to centers with different infrastructures or surgical expertise. Multicenter studies with broader practice variation are needed to confirm the generalizability of our observations.

**Figure 3.**
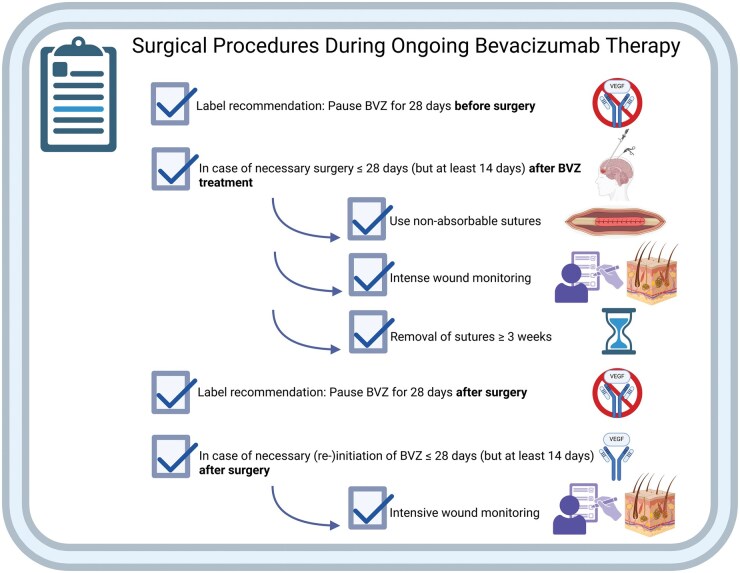
Recommended workflow. Recommended clinical workflow for Bevacizumab administration in relation to surgical interventions in pediatric CNS tumor patients, highlighting timing considerations, surgical precautions, and postoperative monitoring to optimize wound healing and patient satiety. * BVZ = Bevacizumab. Created in https://BioRender.com.

In summary, our analysis indicates that shorter intervals between BVZ administration and surgical procedures are generally feasible. Surgical revisions, when necessary, were minor and did not substantially affect oncological outcomes. Our results support the feasibility of shorter cessation intervals for BVZ when oncological priorities demand expedited surgical or therapeutic interventions, provided that appropriate surgical techniques and vigilant wound monitoring are employed (see Figure 3).

## Conclusion

In this study, we investigated perioperative wound complications associated with Bevacizumab administration in pediatric patients with CNS tumors undergoing surgical interventions. Our findings indicate that BVZ can be administered in the majority of cases ≤ 28 days, both before and after surgery, provided that non-absorbable sutures are employed. However, considering the slightly elevated risk of wound complications, rigorous postoperative monitoring and timely management of any signs of wound impairment remain essential.

## Supplementary Material

vdag073_Supplementary_Data

## Data Availability

Data of this study are available from the corresponding author upon reasonable request.
