# Supplementary material for: Impact of Bevacizumab treatment intervals on surgical interventions in pediatric neuro-oncology
Source: Neurooncol Adv. 2026 Mar 21;8(1):vdag073. doi: 10.1093/noajnl/vdag073 (PMC13110871; doi:10.1093/noajnl/vdag073)
Supplement: vdag073_Supplementary_Data [file vdag073_supplementary_data.docx]

## Supplementary Table and Figure Legends

**Tables**

*Table S1: Tumor Entities*

Overview of all tumor entities included in the study cohort.

*Table S2: Surgical Procedures*

Summary of all surgical interventions, including classification into minor and major procedures.

**Figures**

*Figure S1: Tumor Entities*

Distribution of tumor entities across the study population.

Table S1: Tumor Entities

| **Parameter** | **Unit** | **Amount** |
| --- | --- | --- |
| **Tumor entities** |  |  |
| Medulloblastoma | n (%) | 35/119 (29.41) |
| Low-grade glioma (LGG) | n (%) | 24/119 (20.17) |
| Ependymoma | n (%) | 15/119 (12.61) |
| High-grade glioma (HGG) | n (%) | 14/119 (11.76) |
| Atypical teratoid/rhabdoid tumor (ATRT) | n (%) | 12/119 (10.08) |
| Embryonal tumor with multi-layered rosettes (ETMR) | n (%) | 3/119 (2.52) |
| Malignant peripheral nerve sheath tumor (MPNST) | n (%) | 3/119 (2.52) |
| Primitive neuroectodermal tumor (PNET, *currently under reclassification*) | n (%) | 2/119 (1.68) |
| Meningioma | n (%) | 2/119 (1.68) |
| Astroblastoma | n (%) | 1/119 (0.84) |
| Chordoma | n (%) | 1/119 (0.84) |
| Choroid plexus carcinoma | n (%) | 1/119 (0.84) |
| Craniopharyngioma | n (%) | 1/119 (0.84) |
| Epithelioid sarcoma | n (%) | 1/119 (0.84) |
| High-grade neuroepithelial tumor with BCOR alteration (HGNET BCOR) | n (%) | 1/119 (0.84) |
| Neuroblastoma | n (%) | 1/119 (0.84) |
| Paraganglioma | n (%) | 1/119 (0.84) |
| Pineoblastoma | n (%) | 1/119 (0.84) |
|  |  |  |

Table S2: Surgical Procedures

| **Surgical procedures during BVZ treatment** | **Amount** | **Surgery type** |
| --- | --- | --- |
|  |  |  |
| ***Minor non-neurosurgical procedures (n = 136)*** | | |
| Implantation of a central venous catheter (PAC, Broviac® or Hickman®) | 81 | 1 |
| Revision of a central venous catheter (PAC, Broviac® or Hickman®) due to mechanical dysfunction | 12 | 1 |
| Explantation of a central venous catheter (PAC, Broviac® or Hickman®) as the catheter is no longer needed | 8 | 1 |
| Implantation of percutaneous endoscopic gastrostomy (PEG) tube | 7 | 1 |
| Revision or explantation of a central venous catheter (PAC, Broviac® or Hickman®) due to positive germ in central blood culture | 5 | 1 |
| Dental surgery (e.g. tooth extraction) | 3 | 1 |
| Combined explantation and implantation of a central venous catheter (PAC, Broviac® or Hickman®) | 3 | 1 |
| Thoracic drainage | 3 | 1 |
| Ophthalmologic surgery (e.g. amniotic cover and tarsorrhaphy, combined strabismus surgery) | 3 | 1 |
| Urological surgery (e.g. installation of a percutaneous bladder catheter, circumcision) | 2 | 1 |
| ENT surgery (e.g. paracentesis & adenotomy) | 2 | 1 |
| Biopsy (sternal tumor mass, lung) | 2 | 1 |
| Bone marrow aspiration | 1 | 1 |
| Extirpation of plastic part after PAC explantation | 1 | 1 |
| Combined PAC and PEG implantation | 1 | 1 |
| Coloscopy with polyp removal | 1 | 1 |
| ENT surgery (tracheostomy) | 1 | 1 |
| ***Major non-neurosurgical procedures (n = 9)*** | | |
| Laparotomy (e.g. with pancreatic tail resection, splenectomy removal of a retroperitoneal mass / with ulcer suturing) | 3 | 2 |
| Orthopedic surgery | 2 | 2 |
| Oral and maxillofacial surgery | 2 | 2 |
| Decubitus debridement | 1 | 2 |
| Lymph node extirpation | 1 | 2 |
| ***Minor neurosurgical procedures (n = 120)*** | | |
| Implantation of Ommaya®-reservoir | 38 | 3 |
| Implantation of ventricular drainage (VP-shunt, SD-shunt, EVD) | 29 | 3 |
| Revision of Ommaya®-reservoir or ventricular drainage (VP-shunt, SD-shunt, EVD) due to mechanical dysfunction | 20 | 3 |
| Biopsy (open, stereotactic) | 18 | 3 |
| Revision of Ommaya®-reservoir or ventricular drainage (VP-shunt, SD-shunt, EVD) due to positive germ in cerebrospinal fluid | 6 | 3 |
| Implantation of Ommaya®-reservoir & implantation of VP-shunt or on/off valve for VP-shunt | 5 | 3 |
| Implantation of VP-shunt & biopsy | 2 | 3 |
| Removal of small intracerebral metal splinters | 1 | 3 |
| Borehole trepanation with implantation of a Jackson-Pratt Drain | 1 | 3 |
| ***Major neurosurgical procedures (n = 79)*** | | |
| Resection of tumor or metastasis | 75 | 4 |
| Resection of tumor & implantation of Ommaya®-reservoir | 2 | 4 |
| Resection of metastasis & implantation of VP-shunt | 2 | 4 |
|  |  |  |

ENT = ear, nose, throat; EVD = extra-ventricular drainage; PAC = Port-a-Cath; PEG = percutaneous endoscopic gastrostomy; SD-shunt = subduroperitoneal shunt; VP-shunt = ventriculoperitoneal shunt; 1 = minor non-neurosurgery; 2 = major non-neurosurgery; 3 = minor neurosurgical procedure; 4 = major neurosurgical procedure.

Figure S1: Tumor Entities


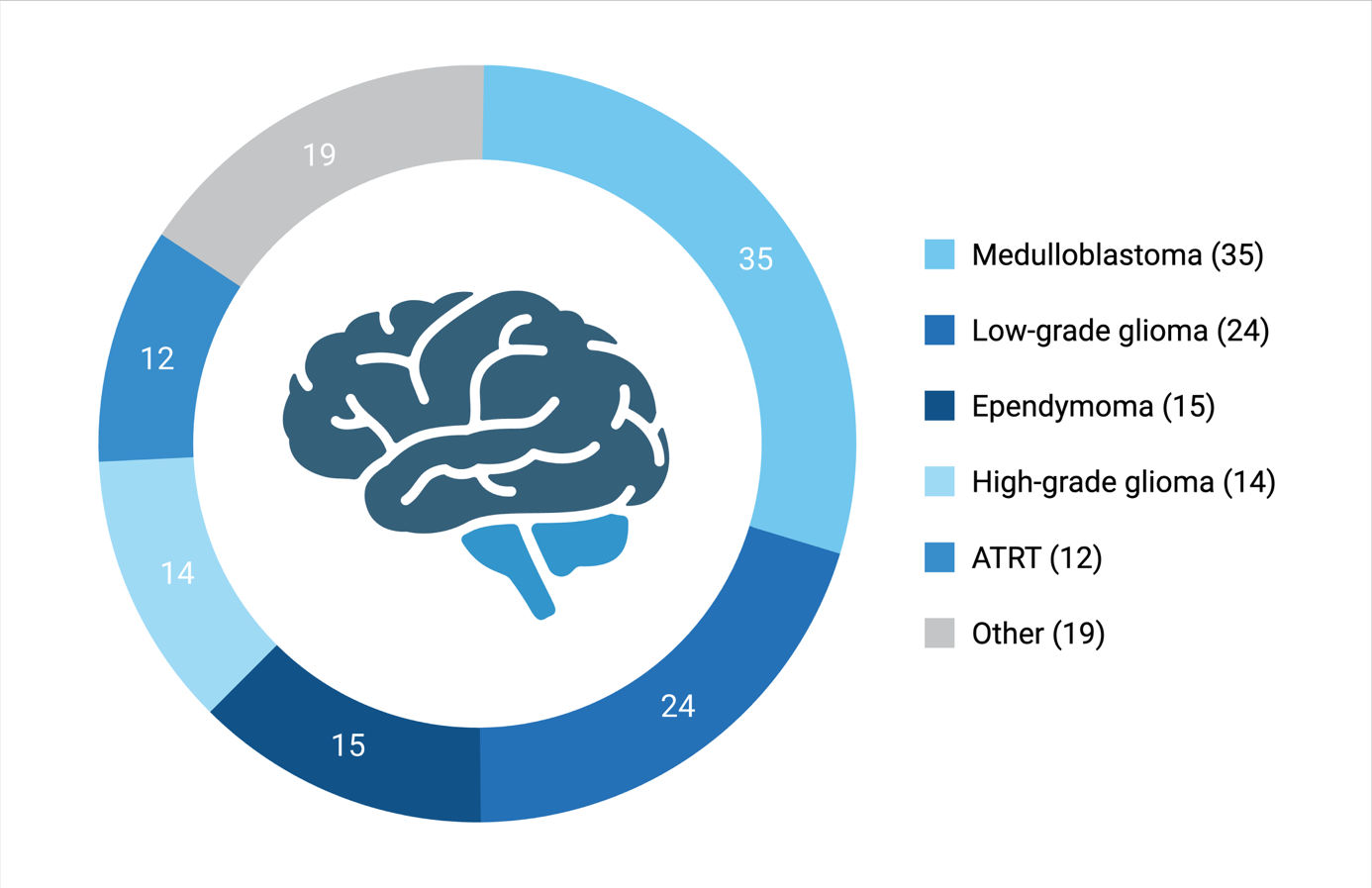


Tumor entities of included patients (n = 119). **Medulloblastoma n = 35 (29.41 %)**; **low-grade glioma n = 24 (20.17 %)** [pilocytic astrocytoma n = 22, diffuse low-grade glioma n = 1, xanthoastrocytoma n = 1]; **ependymoma n = 15 (12.61 %)**; **high-grade glioma n = 14 (11.76 %)** [diffuse midline glioma, H3K27M-mutated n = 11, glioblastoma IDH-wildtype n = 2, diffuse pediatric-type high-grade glioma, H3-wildtype and IDH-wildtype n = 1]; **ATRT n = 12 (10.08 %)**; **other n = 19** **(15.97 %)** [PNET n = 3 (*currently not yet otherwise classified*), ETMR n = 3, malignant peripheral nerve sheath tumor n = 2, meningioma n = 2, astroblastoma n = 1, chordoma n = 1, choroid plexus carcinoma n = 1, craniopharyngioma n = 1, epitheloid sarcoma n = 1, high-grade neuroepithelial tumor with BCOR alteration (HGNET BCOR) n = 1, neuroblastoma n = 1, paraganglioma n = 1, pineoblastoma n = 1]. Created in https://BioRender.com.
